# The Association Between Borderline Dysnatremia and Perioperative Morbidity and Mortality: Retrospective Cohort Study of the American College of Surgeons National Surgical Quality Improvement Program Database

**DOI:** 10.2196/38462

**Published:** 2023-03-16

**Authors:** Jacob H Cole, Krista B Highland, Scott B Hughey, Brendan J O'Shea, Thomas Hauert, Ashton H Goldman, George C Balazs, Gregory J Booth

**Affiliations:** 1 Department of Anesthesiology, Perioperative, and Pain Medicine Brigham and Women's Hospital Boston, MA United States; 2 Department of Anesthesiology Uniformed Services University Bethesda, MD United States; 3 Naval Biotechnology Group Naval Medical Center Portsmouth Portsmouth, VA United States; 4 Department of Anesthesiology and Pain Medicine Naval Medical Center Portsmouth Portsmouth, VA United States; 5 Department of Orthopedic Surgery Naval Medical Center Portsmouth Portsmouth, VA United States

**Keywords:** hypernatremia, hyponatremia, perioperative care, postoperative complications, reference values, sodium, morbidity, mortality, database, data, cohort, surgery, sodium, preoperative, serum

## Abstract

**Background:**

Hyponatremia and hypernatremia, as conventionally defined (<135 mEq/L and >145 mEq/L, respectively), are associated with increased perioperative morbidity and mortality. However, the effects of subtle deviations in serum sodium concentration within the normal range are not well-characterized.

**Objective:**

The purpose of this analysis is to determine the association between borderline hyponatremia (135-137 mEq/L) and hypernatremia (143-145 mEq/L) on perioperative morbidity and mortality.

**Methods:**

A retrospective cohort study was performed using data from the American College of Surgeons National Surgical Quality Improvement Program database. This database is a repository of surgical outcome data collected from over 600 hospitals across the United States. The National Surgical Quality Improvement Program database was queried to extract all patients undergoing elective, noncardiac surgery from 2015 to 2019. The primary predictor variable was preoperative serum sodium concentration, measured less than 5 days before the index surgery. The 2 primary outcomes were the odds of morbidity and mortality occurring within 30 days of surgery. The risk of both outcomes in relation to preoperative serum sodium concentration was modeled using weighted generalized additive models to minimize the effect of selection bias while controlling for covariates.

**Results:**

In the overall cohort, 1,003,956 of 4,551,726 available patients had a serum sodium concentration drawn within 5 days of their index surgery. The odds of morbidity and mortality across sodium levels of 130-150 mEq/L relative to a sodium level of 140 mEq/L followed a nonnormally distributed U-shaped curve. The mean serum sodium concentration in the study population was 139 mEq/L. All continuous covariates were significantly associated with both morbidity and mortality (*P*<.001). Preoperative serum sodium concentrations of less than 139 mEq/L and those greater than 144 mEq/L were independently associated with increased morbidity probabilities. Serum sodium concentrations of less than 138 mEq/L and those greater than 142 mEq/L were associated with increased mortality probabilities. Hypernatremia was associated with higher odds of both morbidity and mortality than corresponding degrees of hyponatremia.

**Conclusions:**

Among patients undergoing elective, noncardiac surgery, this retrospective analysis found that preoperative serum sodium levels less than 138 mEq/L and those greater than 142 mEq/L are associated with increased morbidity and mortality, even within currently accepted “normal” ranges. The retrospective nature of this investigation limits the ability to make causal determinations for these findings. Given the U-shaped distribution of risk, past investigations that assume a linear relationship between serum sodium concentration and surgical outcomes may need to be revisited. Likewise, these results question the current definition of perioperative eunatremia, which may require future prospective investigations.

## Introduction

Abnormal preoperative sodium levels are associated with multiple adverse outcomes, including increased risk of venous thromboembolism, major bleeding and return to the operating room, perioperative coronary events, wound infection, and prolonged postoperative length of hospital stay [1-6]. Both hyponatremia and hypernatremia are associated with an increased risk of perioperative mortality [2,4,5]. Past investigations in nonsurgical populations suggest that optimizing sodium intake may reduce the risk of mortality [7,8]. While these studies provide a clinical rationale for intervention in the presence of hyponatremia or hypernatremia, the granularity of results has been limited due to broad categorizations of hyponatremia and hypernatremia.

Many previous studies investigating patient outcomes categorize sodium levels as hyponatremic (serum sodium concentration less than 135 mEq/L), eunatremic, and hypernatremic (serum sodium concentration greater than 145 mEq/L) [1,9-11]. Some studies also identified an increased risk of in-hospital and 1-year mortality in hospitalized patients with mild hyponatremia (125-134 mEq/L) and hypernatremia (146-150 mEq/L) [12,13]. Such evidence indicates that there are gradations of risk per sodium level *outside* of the eunatremic range, but it is unknown if such gradations of risk occur *within* the eunatremic range. Therefore, a more granular resolution is needed to determine if there is an increased risk of poor postoperative outcomes in patients within the range of serum sodium concentrations that are currently accepted as normal.

The culmination of research to date indicates that the role of sodium in morbidity and mortality risk is broad across a variety of surgeries, including hip arthroplasty [7,8], lower extremity arthroplasty [14], cervical spinal fusion [15], and cardiac surgery [9,16]. Moreover, risk prediction models, including those based on the American College of Surgeons National Surgical Quality Improvement Program (ACS NSQIP) data, indicate that sodium level, when categorized (eg, hyponatremia, eunatremic, and hypernatremia), is an important indicator of postsurgical morbidity and mortality in a large surgically diverse sample [17]. Such risk models do not allow clinicians to delineate an ideal target for clinical intervention. Taken together, there is a need to provide clinically informative research that evaluates the nonnormally distributed relationship between sodium levels, morbidity, and mortality across a large surgical population. Therefore, the purpose of this investigation was to explore the potential nonlinear relationship between preoperative sodium levels, modeled as a continuous predictor, and the odds of 30-day postoperative morbidity and mortality in patients undergoing elective, noncardiac surgery. We hypothesized that preoperative serum sodium concentration was independently associated with increased odds of both postoperative morbidity and mortality when modeled as a continuous variable, assuming a reference normal serum sodium concentration of 140 mEq/L.

## Methods

### Ethical Considerations

This study is a retrospective cohort design and was approved by the Naval Medical Center Portsmouth’s Institutional Review Board (NMCP.2021.0054).

### Study Design and Data Source

Data from the ACS NSQIP database during the years 2015-2019 were obtained. These data come from over 700 hospitals and are collected using well-described methods to assure a high level of validity [18]. Noncardiac surgical procedures were included using current procedural terminology (CPT) codes 10000-32999 and 34000-69999. Patients undergoing cardiac surgery were excluded from this analysis due to the unique risks associated with that patient population, including the risks associated with cardiopulmonary bypass. Similar to previous investigations [19], we excluded minor surgeries such as endoscopies (CPT 43200-43272, 45300-45392, 46600-46608) and minor musculoskeletal procedures (CPT 29000-29750). Additionally, patients were excluded if they underwent emergency surgery.

The following demographic and health data were collected for each patient: CPT code, age, race, ethnicity, height, weight, sex assigned in the medical record, functional status, American Society of Anesthesiologists (ASA) Physical Score, sodium level, hematocrit, creatinine, steroid use, ascites, sepsis or septic shock, ventilator dependence, disseminated cancer, diabetes, hypertension, weight loss (at least 10% in the past year), congestive heart failure (CHF), dyspnea, smoking, chronic obstructive pulmonary disease, and dialysis. Patient records were included based on the following criteria: sodium, hematocrit, and creatinine assessment <5 days prior to surgery; BMI of >12 and <60; ages 18 to 89 years; hematocrit of >21% and <50%; sodium level of ≥130 mEq/L and ≤150 mEq/L; creatinine level of ≥0.5 mg/dL and ≤4.0 mg/dL; and undergoing surgery under a primary CPT listed in at least 50 patient records.

### Exposure

The primary exposure was the preoperative sodium level. *A priori*, the serum sodium level of 140 mEq/L was empirically determined to be the reference value for the development of statistical models.

### Outcomes

The 2 primary outcomes were defined as aggregate morbidity within 30 days of index surgery and mortality within 30 days of index surgery. Aggregate morbidity included any of the following: cardiac arrest, myocardial infarction, cerebrovascular accident, deep vein thrombosis, pulmonary embolism, postoperative sepsis or septic shock, renal insufficiency or failure, reintubation, failure to wean from the ventilator, pneumonia, wound dehiscence, or surgical site infection (including superficial, deep, or organ space). Details regarding the standardized definitions of these variables have been previously published [19].

### Statistical Analysis

#### Univariate and Bivariate Analyses

First, nonparametric analyses (eg, chi-square, Kruskal-Wallis rank sum, and Mann-Whitney U tests) examined differences between patient records that were and were not included in the analyses. Next, bivariate analyses evaluated differences in demographic characteristics and medical comorbidities by morbidity and mortality status. Bivariate analyses were performed using the *TableOne* R package (R Foundation) [20]. Due to the elevated likelihood of rejecting the null hypothesis (*P*<.05) in large samples and because the information rendered by the *P* value does not describe the strength of differences, both the *P* value and the standardized mean difference are reported for bivariate analyses. Standardized mean difference is reported specifically to describe the effect size of the included demographic characteristics and medical comorbidities on the outcomes of morbidity and mortality.

#### Inverse Probability Weights

Given the potential for selection bias in this analysis, outcome models included weights corresponding to the inverse probability of meeting inclusion criteria. This previously validated method accounts for selection bias due to missing predictor data [21]. Inverse probability weights were constructed through a multistep process. First, a generalized additive model (GAM) was conducted using the *mgcv* R package [22] to estimate the propensity of record inclusion. GAMs allowed for the modeling of nonlinear relationships between continuous predictors and the outcomes (smooth effects). In the GAM, the binary outcome was recorded as exclusion (0) versus inclusion (1), and the predictors were covariates associated with included versus excluded status. Sodium, creatinine, and hematocrit were not used in this analysis, as the lack of preoperative laboratory data was indicative of an excluded status. To account for the role of primary CPT in the propensity to be included, the proportion (%) of included patients per primary CPT was calculated. This proportion was included in the GAM as an additional covariate. The predicted and fitted values indicated the propensity of record inclusion given demographic characteristics, medical comorbidities, and primary CPT. Lastly, the propensity scores were transformed into inverse probability weights through the following formula: Inverse probability weight = (Included status / Propensity score) + ((1 Included status) / (1 Propensity score)). These weights were used to control for potential selection bias in subsequent outcome models [23].

#### Generalized Additive Models

The previously described factors associated with morbidity and mortality within the NSQIP database were included as covariates in 2 separate GAMs. One model was generated to predict aggregate morbidity, and the other to predict mortality. If missing data in the included sample was >1%, multiple imputations were planned. To assess the degree of multicollinearity, the *performance* R package was used to compute the variance inflation factor of each fixed covariate; a variance inflation factor <5 indicated acceptable levels of multicollinearity. GAM results were extracted using the *sjPlot* R package [24]. Estimated conditional means (95% CI) were calculated using the *ggeffects* R package [25]. Both the adjusted odds ratios (95% CI) and adjusted relative risks (RRs, 95% CI) of morbidity and mortality at sodium levels 130-150 mEq/L, relative to the *a priori* defined reference of 140 mEq/L, were calculated as well. The *ggplot2* [26] and *ggpubr* [27] R packages were used to construct customized plots of model results. Statistical significance was indicated by *P*<.05.

#### Sensitivity Analysis

Sensitivity analyses were performed using E-values [28] and stratification of the included sample by the previously calculated propensity scores. The *EValue* R package [29] was used to calculate E-values corresponding to each RR of sodium levels 130-150 mEq/L. E-values indicate the strength a confounding variable would need to have on both the predictor (sodium) and outcome, beyond the effects of covariates already included in the model, to render the effect of sodium on the outcome null [30]. As such, E-values provide an assumption-free means of evaluating the robustness of model results [28]. For comparison purposes, the RR (95% CI) of fixed effects was also calculated. Within the included sample, propensity scores corresponding to the propensity to be included in analyses were divided into terciles. The outcome GAMs were replicated without the weights in the subsample of included records with the lowest tercile of propensity scores. Sensitivity analyses were graphically rendered for comparison purposes.

## Results

### Sample Description

Of the 4,551,726 patient records available, 1,003,956 met all inclusion criteria. Most patient records were excluded due to laboratory assessments occurring more than 4 days from surgery or not at all (n=3,388,178), continuous variables outside of the prespecified ranges (n=145,458), and a primary CPT that was not represented in at least 50 patient records (n=14,134). Bivariate analyses indicated that those included versus excluded differed across all identified demographic characteristics and medical comorbidities (Multimedia Appendix 1). In the included sample, 15,474 (0.3%) patient records had missing data; therefore, no imputation was performed. Morbidity and mortality rates in the included cohort were 8.5% and 1.3%, respectively. Descriptive statistics are reported (Table 1). Morbidity (Table 2) and mortality status (Table 3) are also reported. Bivariate test results indicated that all demographic characteristics and medical comorbidities were associated with morbidity and mortality status. As such, all of these factors were included as covariates in the GAMs.

**Table 1:** Descriptive statistics of the overall sample (N=977,343).

Characteristics			Overall
Age (years), median (IQR)			60.0 (46.0-71.0)
**Sex, n (%)**			
	Male	452,054 (45.0)	
	Female	551,884 (55.0)	
**Race and ethnicity, n (%)**			
	White	654,377 (65.2)	
	American Indian and Alaska Native	6366 (0.6)	
	Asian	27,927 (2.8)	
	Black	111,166 (11.1)	
	Latino	73,748 (7.3)	
	Native Hawaiian and Pacific Islander	3575 (0.4)	
	Other	2451 (0.2)	
	Unknown	124,346 (12.4)	
BMI, median (IQR)			28.66 (24.69-33.67)
**ASA^a^ physical status, n (%)**			
	I	56,585 (5.7)	
	II	387,503 (38.7)	
	III	477,321 (47.7)	
	IV	79,712 (8.0)	
**Presence of comorbidities, n (%)**			
	Hypertension	481,752 (48.0)	
	Diabetes	191,078 (19.6)	
	COPD^b^	56,487 (5.6)	
	History of smoking	200,591 (20.0)	
	Chronic steroid use	48,421 (4.8)	
	Congestive heart failure	14,385 (1.4)	
	Active cancer diagnosis	40,880 (4.1)	
	Sepsis or septic shock	37,231 (3.8)	
**Preoperative laboratory values, median (IQR)**			
	Sodium (mEq/L)	139 (137-141)	
	Hematocrit (%)	39.2 (35.2-42.5)	
	Creatinine (mg/dL)	0.84 (0.70-1.01)	
Percent CPT^c^ morbidity (IQR)			5.30 (2.63-11.28)
Percent CPT mortality (IQR)			0.24 (0.08-1.47)

^a^ASA: American Society of Anesthesiologists.

^b^COPD: chronic obstructive pulmonary disease.

^c^CPT: current procedural terminology.

**Table 2:** Aggregate morbidity outcomes status.

Characteristics			No morbidity (N=918,385)		Morbidity (N=85,571)		*P* value			SMD^a^	
Age (years), median (IQR)			59.0 (45.0-70.0)		65.0 (54.0-75.0)		<.001			0.38	
**Sex, n (%)**								<.001			0.08
	Male	410,367 (44.7)		41,687 (48.7)							
	Female	508,002 (55.3)		43,882 (51.3)							
**Race and ethnicity, n (%)**								<.001			0.1
	White	597,599 (65.1)		56,778 (66.4)							
	American Indian and Alaska Native	5814 (0.6)		552 (0.6)							
	Asian	25,943 (2.8)		1984 (2.3)							
	Black	100,829 (11.0)		10,337 (12.1)							
	Latino	69,192 (7.5)		4556 (5.3)							
	Native Hawaiian and Pacific Islander	3303 (0.4)		272 (0.3)							
	Other	2281 (0.2)		170 (0.2)							
	Unknown	113,424 (12.4)		10,922 (12.8)							
BMI, median (IQR)			28.69 (24.74-33.67)		28.69 (24.74-33.67)		<.001			0.03	
**ASA^b^ physical status, n (%)**								<.001			0.57
	I	55,391 (6.0)		1194 (1.4)							
	II	369,550 (40.3)		17,953 (21.1)							
	III	426,405 (46.6)		50,916 (59.8)							
	IV	64,588 (7.1)		15,124 (17.8)							
**Presence of comorbidities, n (%)**											
	Hypertension	430,631 (46.9)		51,121 (59.7)		<.001			0.26		
	Diabetes	168,658 (18.4)		22,420 (26.2)		<.001			0.21		
	COPD^c^	47,235 (5.1)		9252 (10.8)		<.001			0.21		
	History of smoking	181,440 (19.8)		19,151 (22.4)		<.001			0.06		
	Chronic steroid use	41,464 (4.5)		6957 (8.1)		<.001			0.15		
	Congestive heart failure	11,224 (1.2)		3161 (3.7)		<.001			0.16		
	Active cancer diagnosis	33,660 (3.7)		7220 (8.4)		<.001			0.20		
	Sepsis or septic shock	31,324 (3.4)		5907 (6.9)		<.001			0.17		
**Preoperative laboratory values, median (IQR)**											
	Sodium (mEq/L)	139 (137-141)		139 (137-141)		<.001			0.12		
	Hematocrit (%)	39.4 (35.6-42.6)		37.0 (32.0-41.0)		<.001			0.41		
	Creatinine (m)g/dL	0.83 (0.70-1.00)		0.88 (0.70-1.11)		<.001			0.22		
Percent CPT^d^ morbidity (IQR)			5.02 (2.63-10.26)		12.38 (5.93-19.54)		<.001			0.81	
Percent CPT mortality (IQR)			0.22 (0.08-1.15)		1.25 (0.33-3.05)		<.001			0.51	

^a^SMD: standardized mean difference.

^b^ASA: American Society of Anesthesiologists.

^c^COPD: chronic obstructive pulmonary disease.

^d^CPT: current procedural terminology.

**Table 3:** Mortality outcome status.

Characteristics		No mortality (N=991,327)	Mortality (N=12,629)	*P* value	SMD^a^
Age (years), median (IQR)		60.00 (46.00-71.00)	75.00 (66.00-82.00)	<.001	1.02
**Sex, n (%)**				<.001	0.19
	Male	445,213 (44.9)	6841 (54.2)		
	Female	546,096 (55.1)	5788 (45.8)		
**Race and ethnicity, n (%)**				<.001	0.24
	White	645,010 (65.1)	9367 (74.2)		
	American Indian & Alaska Native	6315 (0.6)	51 (0.4)		
	Asian	27,682 (2.8)	245 (1.9)		
	Black	109,799 (11.1)	1367 (10.8)		
	Latino	73,256 (7.4)	492 (3.9)		
	Native Hawaiian and Pacific Islander	3546 (0.4)	29 (0.2)		
	Other	2430 (0.2)	21 (0.2)		
	Unknown	123,289 (12.4)	1057 (8.4)		
BMI, median (IQR)		28.69 (24.74-33.73)	25.99 (22.20-30.99)	<.001	0.35
**ASA^b^ physical status, n (%)**				<.001	1.25
	I	56,574 (5.7)	11 (0.1)		
	II	386,890 (39.1)	613 (4.9)		
	III	470,707 (47.6)	6614 (53.2)		
	IV	74,518 (7.5)	5194 (41.8)		
**Presence of comorbidities, n (%)**					
	Hypertension	472,832 (47.7)	8920 (70.6)	<.001	0.48
	Diabetes	187,320 (18.9)	3758 (29.8)	<.001	0.29
	COPD^c^	54,107 (5.5)	2380 (18.8)	<.001	0.42
	History of smoking	198,133 (20.0)	2458 (19.5)	.15	0.01
	Chronic steroid use	47,044 (4.7)	1377 (10.9)	<.001	0.23
	Congestive heart failure	13,072 (1.3)	1313 (10.4)	<.001	0.39
	Active cancer diagnosis	38,451 (3.9)	2429 (19.2)	<.001	0.50
	Sepsis or septic shock	35,342 (3.6)	1889 (1.5)	<.001	0.42
**Preoperative laboratory values, median (IQR)**					
	Sodium (mEq/L)	139 (137-141)	138 (136-141)	<.001	0.19
	Hematocrit (%)	39.3 (35.3-42.5)	33.0 (28.8-38.0)	<.001	0.88
	Creatinine (mg/dL)	0.83 (0.70-1.01)	1.00 (0.76-1.44)	<.001	0.54
Percent CPT^d^ morbidity (IQR)		5.30 (2.63-10.90)	12.54 (9.08-18.46)	<.001	0.91
Percent CPT mortality (IQR)		0.23 (0.08-1.37)	2.91 (1.25-5.09)	<.001	1.05

^a^SMD: standardized mean difference.

^b^ASA: American Society of Anesthesiologists.

^c^COPD: chronic obstructive pulmonary disease.

^d^CPT: current procedural terminology.

### GAM Results

**Figure 1:**
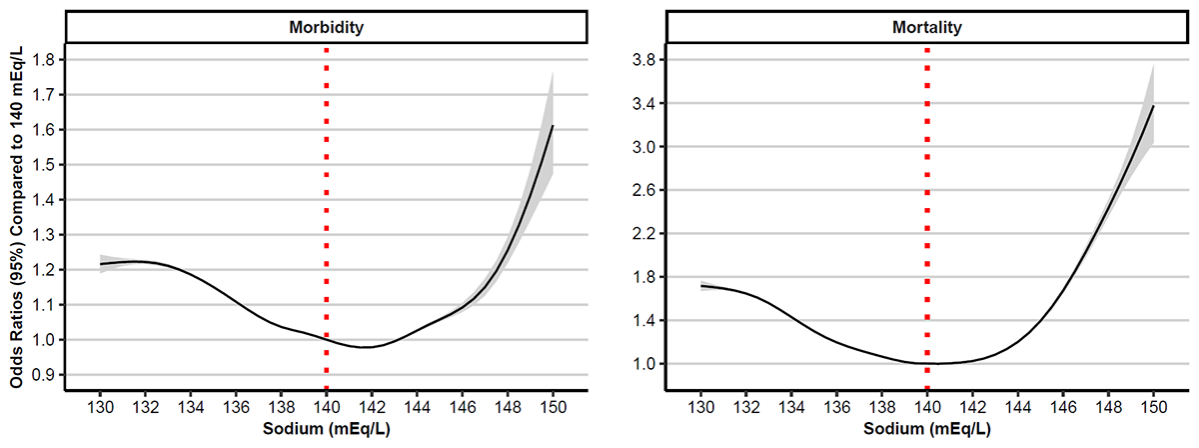
Odds ratios (95% CI) of morbidity (left) and mortality (right).

In both outcome GAMs, all continuous covariates (age, BMI, sodium, hematocrit, creatinine, and percent CPT morbidity or mortality) were modeled as smooth terms and were substantially associated with both morbidity and mortality. Across both models, patients assigned male in the medical record with an elevated ASA status, steroid use, sepsis or septic shock, cancer, a positive smoking status, chronic obstructive pulmonary disease, renal failure, and CHF were more likely to experience morbidity and mortality compared to their reference counterparts (Multimedia Appendix 2). When controlling for other demographic characteristics and medical comorbidities, patients whose race and ethnicity were listed as Asian or Latino had a lower probability of morbidity and mortality relative to White patients. Similarly, White patients had a greater probability of morbidity relative to Native Hawaiian and Pacific Islander patients, but White patients had a lower probability of morbidity than patients of unknown race and ethnicity. Patients with diabetes and hypertension had a greater probability of morbidity relative to those without these conditions. The odds of morbidity and mortality across sodium levels of 130-150 mEq/L relative to a sodium level of 140 mEq/L followed a U-shaped curve (Figure 1).

### Sensitivity Analyses

The E-values corresponding to the RR of morbidity and mortality across sodium levels are shown in Figures S1A and S1B in Multimedia Appendix 3. For example, at a sodium level of 135 mEq/L, the morbidity (RR 1.07, 95% CI 1.07-1.07) and mortality (RR 1.30, 95% CI 1.30-1.30) would be rendered null if an unmeasured confounder was associated with both sodium and the outcome by a RR of 1.35-fold (lower 95% CI 1.35) and 1.93-fold (lower 95% CI 1.92), respectively. For reference, these E-values are similar to the effects of ASA I versus III on morbidity (RR 1.33, 95% CI 1.32-1.33) and CHF on mortality (RR 1.97, 95% CI 1.94-1.99). The RR (95% CI) for fixed effects on both outcomes are reported in Multimedia Appendix 2.

Lastly, GAMs evaluating morbidity and mortality were conducted on a subsample of the included group with the lowest tercile propensity scores (n=333,701). Model results were similar to the main analysis, such that the effect of sodium was significant (*P*<.001), and the nonlinear pattern followed a U-shape (Figures S1C and S1D in Multimedia Appendix 3).

## Discussion

### Principal Results

This exploratory analysis calls into question the current understanding of the “normal” range of serum sodium levels (135-145 mEq/L) within the context of perioperative care, as values of serum sodium concentration within this range of normal values were associated with 30-day aggregate morbidity and mortality. By examining preoperative sodium levels in over 1 million patients as a continuous variable instead of the commonly used categories (eg, hyponatremic, eunatremic, and hypernatremic), this study provides improved granularity on the association between small deviations in sodium and perioperative outcomes. As such, what is considered “normal” sodium values in the general population may not be normal in patients undergoing elective noncardiac surgery.

### Comparison With Prior Work

As health care shifts to value-based care, these findings may also play a role in evaluating value-based perioperative practices. For example, recent evidence using NSQIP data indicates that preoperative laboratory assessment is not associated with the odds of postoperative complications and readmission in patients undergoing ambulatory surgery with an ASA I or II status, thereby suggesting the low value of preoperative laboratory assessment [31]. However, such findings may be premised on clinician practices that are contingent on a definition of “normal” that is, per these findings, associated with increased risk of aggregate morbidity and mortality (eg, ~135 mEq/L). Given the potential impact of these findings, combined with the lack of causal assumptions that can be made, future work is needed to assess whether clinical intervention addressing high- and low-normal sodium serum concentrations improves clinical outcomes and value-based care.

### Strengths and Limitations

This study possessed several strengths. Though the main variable of interest was serum sodium concentrations, models included many demographic characteristics and medical comorbidities that have previously been shown to be substantially associated with aggregate morbidity and mortality risk. These factors included other laboratory values (eg, creatinine and hematocrit) that may also warrant further inspection, given their relationship with postoperative outcomes. By controlling for these covariates and using a weighted approach based on the inverse probability of record inclusion, the results of this study are likely generalizable to adult patients undergoing any elective, noncardiac surgery in the United States. We restricted records to those with laboratory results collected less than 5 days before surgery, thereby increasing the likelihood that the recorded values actually reflected serum sodium levels at the time of surgery.

This study was tempered by several limitations. First, no causal conclusions can be drawn from the study due to the retrospective, associative nature of the study design and analytic approach. Additionally, there may be several covariates, including specific health conditions, medication receipt (both in the days leading up to surgery and perioperatively), preoperative recommendations (eg, fasting), and prior health care received, that are neither collected in the NSQIP database nor included in the analysis but could be associated with morbidity and mortality. While this database is a robust and extensive collection of surgical outcome data in the United States [18], the inclusion of other covariates mentioned above could serve to refine this model and provide more specific areas of research to explore. Examples of other potential confounders include medications, preoperative fasting, and certain comorbidities, which themselves may be associated with abnormal sodium levels. When considering the potential impact of missing confounders on model results, E-values indicated that any confounder would need to surpass the strength of most fixed covariates within our models and account for unique variance not otherwise accounted for by current covariates to render the effect of sodium null.

### Conclusions

This analysis indicated that both preoperative hyponatremia and preoperative hypernatremia were associated with an increased risk of 30-day aggregate morbidity and mortality. The relationship was nonlinear, such that the risk increased with further deviation from a serum sodium concentration of 140. While prior investigations have demonstrated that dysnatremia is a modifiable risk factor and optimization of preoperative serum sodium levels may represent an opportunity for a reduction in both perioperative morbidity and mortality [31], this study suggests that preoperative serum sodium levels that are within the currently accepted upper and lower limits of normal are likely indicative of elevated risk. As such, future prospective studies are needed to better confer sodium level ranges associated with optimized outcomes after surgery, as well as the potential to directly alter patients’ serum sodium concentrations to improve postoperative outcomes.

## Appendix

Multimedia Appendix 1 Bivariate differences between excluded versus included records.

Multimedia Appendix 2 Risk ratios (95% CI) of fixed covariates across outcomes.

Multimedia Appendix 3 Sensitivity analyses of sodium effects. (A,B) Adjusted relative risk (95%CI) and E-values (lower 95% CI) of morbidity and mortality, respectively. (C,D) Odds ratios (95%CI) of morbidity and mortality, respectively.

## Abbreviations

ACS: American College of Surgeons
ASA: American Society of Anesthesiologists
CHF: congestive heart failure
CPT: current procedural terminology
GAM: generalized additive model
NSQIP: National Surgical Quality Improvement Program
RR: relative risk

## Disclaimer

The views expressed are solely those of the authors and do not reflect the official policy or position of the Uniformed Services University, US Army, US Navy, US Air Force, the Department of Defense, the US Government, or the Henry M Jackson Foundation for the Advancement of Military Medicine, Inc. We are military service members. This work was prepared as part of our official duties. Title 17 U.S.C. 105 provides that “Copyright protection under this title is not available for any work of the United States Government.” Title 17 U.S.C. 101 defines a United States Government work as a work prepared by a military service member or employee of the United States Government as part of that person’s official duties. The American College of Surgeons National Surgical Quality Improvement Program (ACS NSQIP) and the hospitals participating in the ACS NSQIP are the sources of the data used herein; they have not verified and are not responsible for the statistical validity of the data analysis or the conclusions derived by the authors.
