# Supplementary figures and images for: The Association Between Borderline Dysnatremia and Perioperative Morbidity and Mortality: Retrospective Cohort Study of the American College of Surgeons National Surgical Quality Improvement Program Database
Source: JMIR Perioper Med. 2023 Mar 16;6:e38462. doi: 10.2196/38462 (PMC10131592; doi:10.2196/38462)

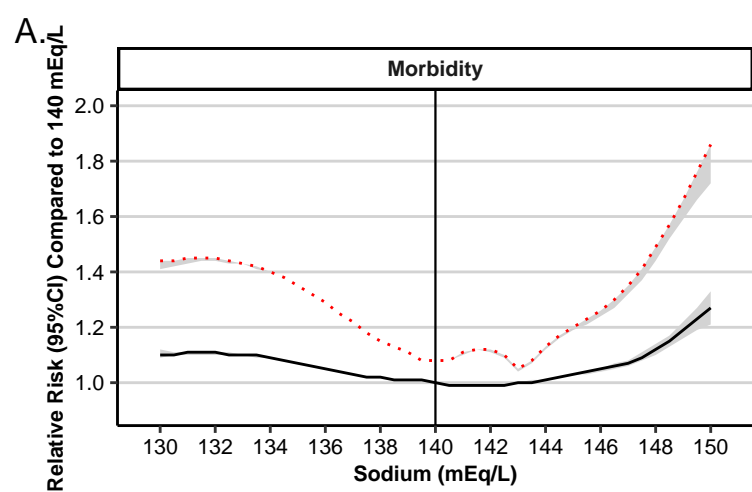

Type: ..... E-Value — Relative Risk

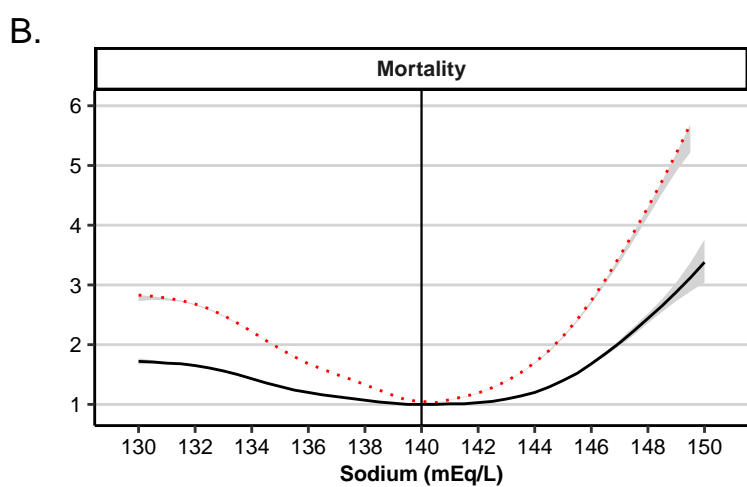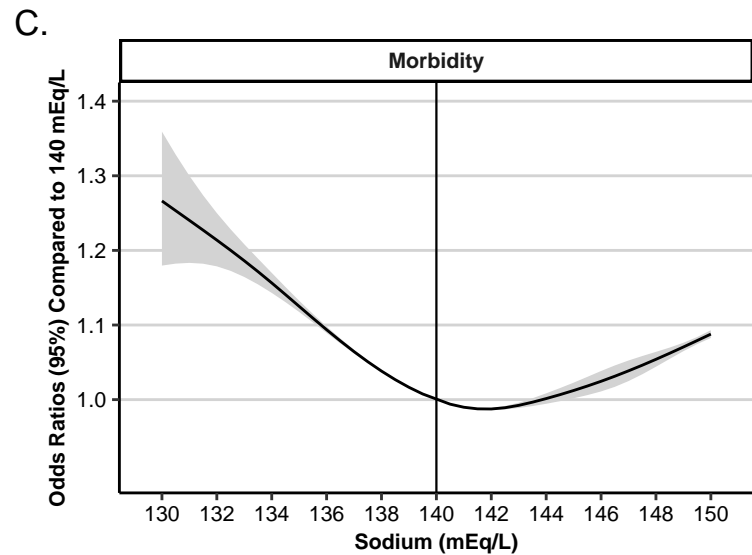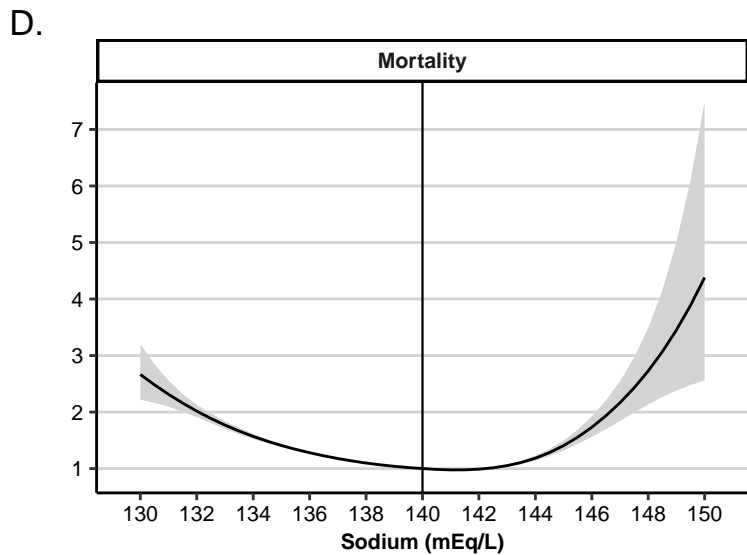

Supplement: Multimedia Appendix 3 [file periop_v6i1e38462_app3.pdf]
